# Barrier protection via Toll-like receptor 2 signaling in porcine intestinal epithelial cells damaged by deoxynivalnol

**DOI:** 10.1186/s13567-016-0309-1

**Published:** 2016-02-09

**Authors:** Min Jeong Gu, Sun Kwang Song, In Kyu Lee, Seongyeol Ko, Seung Eun Han, Suhan Bae, Sang Yun Ji, Byung-Chul Park, Ki-Duk Song, Hak-Kyo Lee, Seung Hyun Han, Cheol-Heui Yun

**Affiliations:** Department of Agricultural Biotechnology and Research Institute for Agriculture and Life Sciences, Seoul National University, Seoul, 151-921 Republic of Korea; Biomodulation Major and Center for Food Bioconvergence, Seoul National University, Seoul, 151-921 Republic of Korea; Biomin Korea Ltd., Seoul, 153-714 Republic of Korea; Seoulfeed Co., Ltd., Incheon, 405-819 Republic of Korea; National Institute of Animal Science, Rural Development Administration, Jeonju, 565-851 Republic of Korea; Institute of Green Bio Science Technology, Seoul National University, Pyeongchang, 232-916 Republic of Korea; Department of Animal Biotechnology, Chonbuk National University, Jeonju, 561-756 Republic of Korea; Department of Oral Microbiology and Immunology, School of Dentistry, Seoul National University, Seoul, 110-749 Republic of Korea

## Abstract

Intestinal barrier is the first line of defense inside the body and comprises intercellular tight junction (TJ) proteins that regulate paracellular permeability. Deoxynivalenol (DON), a fungal metabolite often found in the contaminated food of domestic animals, is known to impair intestinal barrier function and may be involved in intestinal inflammation. Unlike in humans and mice, the importance of Toll-like receptor (TLR) 2 expressed in porcine intestinal epithelial cells is largely unclear. Therefore, the aim of the present study was to investigate whether TLR2 stimulation enhances intestinal barrier function and protects against DON exposure. We found that the cells treated with TLR2 ligands decreased the epithelial barrier permeability and enhanced TJ protein expression in intestinal porcine epithelial cells (IPEC-J2). In addition, pretreatment with TLR2 ligand, including Pam3CSK4 (PCSK) and lipoteichoic acid from *Bacillus subtilis*, prevented DON-induced barrier dysfunction by increasing the expression of TJ proteins via the PI3K-Akt-dependent pathway. It is likely that the DON-disrupted intestinal barrier caused biological changes of immune cells in the lamina propria. Thus, we conducted co-culture of differentiated IPEC-J2 cells in the upper well together with peripheral blood mononuclear cells in the bottom well and found that apical TLR2 stimulation of IPEC-J2 cells could alleviate the reduction in cell survival and proliferation of immune cells. Conclusively, TLR2 signaling on intestinal epithelial cells may enhance intestinal barrier function and prevent DON-induced barrier dysfunction of epithelial cells.

**Electronic supplementary material:**

The online version of this article (doi:10.1186/s13567-016-0309-1) contains supplementary material, which is available to authorized users.

## Introduction

The gastrointestinal tract is chronically exposed to a huge burden of foreign antigens including microorganisms and toxic molecules. Intestinal epithelial cells (IECs) provide the initial line of mucosal host defense in the intestine. Their ability to act as a physical barrier against antigens, to allow selective absorption of nutrients, and to defend against harmful molecules is crucial for maintaining gut immune homeostasis [1]. Paracellular and intercellular transit of molecules in the intestine is modulated by a complex network of tight junction (TJ) and gap junction linking IECs [2]. For instance, the increased epithelial permeability of TJ may initiate and maintain persistent inflammation in intestinal inflammatory diseases.

Toll-like receptor (TLR) 2, a member of the TLR family that is constitutively expressed in IECs, recognizes conserved microbe-associated molecular patterns of both gram-negative and gram-positive bacteria, such as lipoteichoic acid (LTA), lipoarabinomannan, lipoproteins and peptidoglycan (PGN). TLR2 is known to enhance transepithelial resistance of the IEC barrier through apical redistribution of ZO-1 via protein kinase Cα/δ [3]. Moreover, its stimulation efficiently preserves ZO-1-associated barrier integrity of IECs against stress-induced damage, which is critically controlled by the PI3K/Akt-pathway via MyD88 [4]. However, the precise role of TLR2 in intestinal barrier function in pig remains unclear.

Deoxynivalenol (DON) is a mycotoxin produced by *Fusarium* spp., which is prevalent in animal feed [5]. Ingestion of feed contaminated with DON is toxic to many animal species, and pigs are the most sensitive species [6, 7]. It has been suggested that DON targets dividing cells such as IECs and immune cells [8]. DON alters the expression of transcription factors by readily binding to the ribosomes and rapidly activating mitogen-activated protein kinases, and thus appears to affect the expression of a number of molecules, including membrane receptors and cytokines [9]. This mycotoxin is known to modify the production of nitric oxide (NO) or mucin produced by intestinal epithelium [10, 11], and to increase the susceptibility of animals to intestinal infection [12]. Especially, DON suppresses the expression of TJ proteins and, thus, the barrier function of the intestinal epithelium in pigs and humans [13, 14].

The IEC barrier maintains a well-organized structure and communication between IECs and immune cells in the lamina propria [1]. The formation and distribution of TJ significantly enhances IEC barrier function, thus contributing to the protection of the underlying lamina propria from stress, including invasion by harmful antigens. However, the damage caused by exposure to DON may disrupt this interaction, disturbing the intestinal immune system.

Previously, we found that *Bacillus subtilis* and its LTA could protect IPEC-J2 from DON-induced damage [15]. Based on this, we hypothesized that treatment of TLR2 ligands, such as *B. subtilis*-derived LTA, PGN, and synthetic agonist Pam3CSK4, influences the barrier function of IPEC-J2 cells, which may confer a protective effect against DON-induced damage. The objective of the present study was to investigate the mechanism of TLR2-mediated barrier regulation in IPEC-J2 cells.

## Materials and methods

### Cell culture

Non-transformed porcine jejunum epithelial cell line (IPEC-J2; DSMZ) was cultured in the Dulbecco’s modified Eagle medium (DMEM) and Ham’s F-12 medium mixture at one to one (Gibco Life Technologies, Grand Island, USA) supplemented with 5% heat-inactivated fetal bovine serum (FBS), 1% insulin-transferrin-selenium-X (ITS-X) and antibiotics (all from Invitrogen, Grand Island, USA) in an incubator with atmosphere of 5% CO_2_ at 39 °C. During growth and differentiation of the cells, the medium was replaced every 3 days.

### Treatment

IPEC-J2 cell monolayer was treated with 2 μg/mL of DON (Sigma, Missouri, USA) for 24, 48 or 72 h. To evaluate the effect of TLR2 agonists on the barrier function, IPEC-J2 cells were pretreated with 10 μg/mL of LTA from *B. subtilis* (LTA-BS; Invivogen, San Diego, USA), PGN from *B. subtilis* (PGN-BS; Invivogen), Pam3CSK4 (Pam3Cys-SKKKK; Invivogen) or complete medium as a control for 24 h before DON treatment. In some experiments, 10 μg/mL of the PI3K inhibitor LY294002 (Cell signaling, Massachusetts, USA) or 20 μg/mL of anti-TLR2 neutralizing antibody (eBioscience, San Diego, USA) was treated prior to the treatment with TLR2 ligands.

### Measurement of transepithelial electrical resistance

IPEC-J2 cells were grown in 0.3 cm^2^ polyethylene terephthalate membrane insert with 0.4-mm pore (Corning, New York, USA). The cells were differentiated in the insert until reaching >1000 Ω of transepithelial electrical resistance (TEER) and treated with TLR2 ligands and/or DON. TEER was measured every 24 h with epithelial voltohm meter (EVOM2; World Precision Instruments, Sarasota, USA), and the values were expressed as kΩ × cm^2^.

### Porcine peripheral blood cell isolation

Porcine blood samples were obtained from 2 to 6 months old pigs (Landrace–Yorkshire–Duroc) supplied by Animal Farm, Seoul National University (Suwon, Korea). The use of blood was approved by the Institutional Animal Care and Use Committee of Seoul National University (IACUC No., SNU-131126-3). Whole blood was diluted with PBS at a ratio of 1:1, and porcine peripheral blood mononuclear cells (PBMCs) were isolated by density gradient centrifugation (400 × *g* for 25 min without brake) using Ficoll-paque Plus (Amersham Bioscience, Buckinghamshire, UK). PBMCs were suspended in RPMI 1640 medium supplemented with 10% FBS and 1% antibiotics (Invitrogen).

### Transwell co-culture system

IPEC-J2 cells were grown and differentiated in culture media in 0.3 cm^2^ polyethylene terephthalate membrane inserts with 0.4-mm pore (Corning). PBMCs were added basolaterally and 2 μg/mL of DON was treated apically in 100 μL of culture medium. The co-culture system was incubated for 48 h with or without pretreatment with TLR2 ligands at insert.

### MTT [3-(4,5-dimethylthiazol-2-yl)-2,5-diphenyltetrazolium bromide] assay

IPEC-J2 cells, seeded in cultured media in a 96-well culture plate, were treated with DON for 24 and 48 h in the absence or presence of pretreatment with TLR2 ligands. The cells were cultured with medium alone as control. At the end of incubation, 10 μL of MTT (Sigma) solution (5 mg/mL in PBS) was added to each well for 2 h and the media was discarded. Then, 100 μL of DMSO was added to each well and shaken for 5 min to solubilize the formazan formed in the viable cells [16]. Absorbance was measured at 595 nm using a microplate reader, VersaMax (Molecular devices, Sunnyvale, USA). The cell viability (%) was calculated as the percent ratio of absorbance of the samples against the non-treated control medium.

### Western blot analysis

IPEC-J2 cells were treated with DON in the absence or presence of pretreatment with TLR2 ligands, washed with PBS and lysed in a lysis buffer (20 mM Tris–HCl, 150 mM NaCl, 1 mM EDTA, 1% Triton X-100), followed by a quantitation of protein using Micro BCA kit (Thermo, Rockford, USA). For isolation of cytosolic and membrane parts from IPEC-J2 cells, membrane protein extraction kit (Thermo) was used by its instruction. As previously described [17], the same amount of protein extracts was loaded in 10% Tris–glycine polyacrylamide gels and electrophoresed. Then, the proteins were transferred onto a polyvinylidene difluoride (PVDF) microporous membrane for 2 h at 4 °C and blocked with 5% skim milk in TBS-T (20 mM Tris HCl, 100 mM NaCl, 0.05% Tween 20) for 90 min. The blot was incubated with rabbit anti-claudin-3, -occludin or -zonula occludens (ZO)-1 antibodies (Invitrogen), anti-p-AKT, -p-P70S6K, -Akt, -FAK, and -Bcl-2 antibodies (Cell signaling), or mouse anti-β-actin monoclonal IgG1 antibody (Santa Cruz Biotechnology, Grand Island, USA) overnight. Subsequently, the membrane was washed and incubated with goat anti-rabbit or anti-mouse IgG-HRP (Santa Cruz Biotechnology) for 1 h. The target protein was visualized with enhanced chemiluminescence (ECL) system (GE Healthcare, Waukesha, USA), followed by analysis using ChemiDoc XRS (Bio-rad, Hercules, USA).

### Confocal immunofluorescence microscopy

IPEC-J2 cells, treated with or without DON in the absence or presence of pretreatment with TLR2 ligands, were washed, fixed with PBS containing 4% formaldehyde (30 min, room temperature), permeabilized with 0.5% Triton-X-100 in PBS for 3 min, and blocked with 10% FBS (30 min, room temperature). Samples were incubated with rabbit anti-claudin-3, -occludin and -ZO-1 antibodies (Invitrogen), followed by staining with goat anti-rabbit IgG conjugated with Alexa fluor 488 (BD Biosciences, San Jose, USA), and 4′,6-diamidino-2-phenylinodele for nuclei (Immunobioscience, Raleigh, USA). Images were captured using a laser scanning confocal microscope, LSM700 (Carl Zeiss, Jena, Germany).

### Flow cytometry analysis

Porcine PBMCs were harvested, washed with PBS containing 1% FBS and stained with the following mAb at pre-determined optimal concentrations; mouse anti-porcine CD3e (clone PPT3; Southern Biotech, Birmingham, USA), CD4 FITC (clone 74-12-4; BD Biosciences), CD8a PE (clone 76-2-11; BD Biosciences), CD172a (clone 74-22-15; BD Biosciences) and CD163 PE (clone 2A10/11; AbD Serotec, Langford, UK) antibodies. The cells were incubated for 20 min at 4 °C in the dark. To evaluate proliferation, the cells were labeled with 1 μM of CFSE for 15 min at 37 °C, washed twice with plain medium and cultured with IPEC-J2 cells on the transwell plate. After staining, the cells were washed and the expression of surface markers was measured using a flow cytometry (FACSCantoII, BD Biosciences). All the flow cytometric data were analyzed using FlowJo software (Tree Star, California, USA).

### Annexin V/PI analysis

As previously described [18], floating cells were collected and, then, attached cells were washed with PBS and trypsinized for 5 min. Finally, trypsinized cells and floating cells were added together and stained with Annexin V-APC and propidium iodide (PI). The intensity of the markers was examined by flow cytometry (FACSCantoII, BD Biosciences). All flow cytometic data were analyzed by using FlowJo software (Tree Star).

### Real-time PCR

Total RNA was isolated using TRIzol reagent (Invitrogen) according to the manufacturer’s instructions and reverse-transcribed to generate complementary DNA (cDNA) using oligo-dT primers (Bioneer, Daejeon, Korea). The real-time quantitative PCR was carried out using a StepOne Plus real-time PCR system (Applied Biosystems, Foster City, USA). SYBR^®^ Green PCR Master Mix was used according to manufacturer’s specification (Applied Biosystems). The PCR reaction was carried out in 96-well reaction plate with 10 μL SYBR^®^ green PCR master mix, 0.5 μL primers, 1 μL cDNA template and 8 μL nuclease-free H_2_O. The 40 thermal cycles of 2 min at 50 °C, 10 min at 95 °C, 15 s at 95 °C, 30 s at 60 °C, and 30 s at 72 °C were utilized according to the manufacturer’s recommendation. Relative quantification of target genes was calculated using the 2^−ΔΔ*C*t^ method. Target gene expression was normalized to GAPDH mRNA level. The nucleotide sequences of porcine specific primers for TLR2, monocyte chemoattractant protein-1 (MCP-1), granulocyte–macrophage colony stimulating factor (GM-CSF) and glyceraldehyde 3-phosphate dehydrogenase (GAPDH) are shown in Additional file 1.

### Statistical analysis

Statistical analysis (one-way ANOVA with Tukey posttest or two-way ANOVA with Bonferroni posttest) was performed using the GraphPad Prism (version 5.01, GraphPad Software, San Diego, USA). Differences were considered significant if *p* < 0.05.

## Results

### DON disrupted intestinal barrier function in IPEC-J2 cells

**Figure 1:**
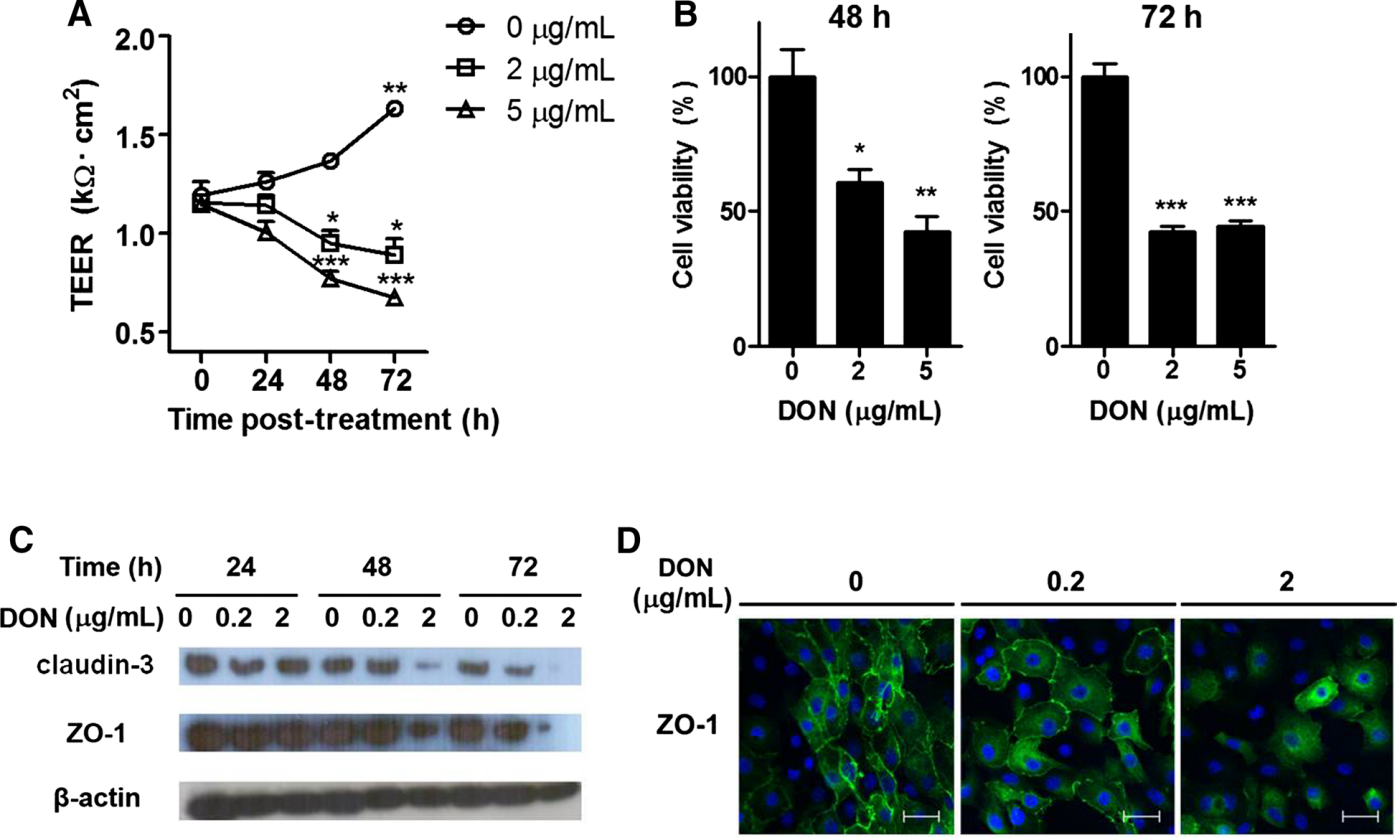
**DON caused intestinal barrier disruption and reduction of IPEC-J2 cell viability.** IPEC-J2 cells were treated with DON (0, 2, or 5 μg/mL) for 0, 24, 48 or 72 h. **A** TEER values were measured using epithelial voltohm meter at indicated time points. Data represent mean ± SD of TEER (*n* = 4). **B** Viability of the cells was examined by MTT assay at 48 and 72 h after DON treatment (*n* = 3). *P < 0.05, **P < 0.01, and ***P < 0.001 001, determined by (**A**) two-way ANOVA with Bonferroni’s posttest, or (**B**) one-way ANOVA with Tukey’s posttest. To examine the expression of tight junction proteins, IPEC-J2 cells were treated with DON (0, 0.2 and 2 μg/mL) for 24, 48, or 72 h. Whole-cell lysates were analyzed for the expression of (**C**) Claudin-3, ZO-1 and β-actin by using Western blot assay. The representative figure from three similar results is shown. **D** ZO-1 expression in IPEC-J2 was visualized using confocal microscopy after staining with anti-ZO-1 antibody conjugated with Alexa fluor 488-FITC (green) and nuclei (DAPI; blue) (*n* = 3). Scale bar = 50 μm.

It has been suggested that DON causes an increase in the permeability of porcine intestinal epithelial cells via mitogen-activated protein kinase signaling [13] by reducing the expression of TJ proteins [19]. In the present study, IPEC-J2 cells treated with DON (0, 2, and 5 μg/mL) for 0, 24, 48, or 72 h showed a time- and dose-dependent reduction in TEER (Figure 1A). We further investigated cell viability after treatment with DON using the MTT assay. The result showed that DON at 2 or 5 μg/mL decreased the viability of IPEC-J2 cells after 48 h and 72 h in a dose-dependent manner (Figure 1B).

The intestinal barrier is interconnected by TJ formed by multi-protein complexes that link adjacent epithelial cells near their apical borders [20]. To investigate the effect of DON on epithelial cells, we examined changes in the expression of TJ proteins (claudin-3 and ZO-1) in IPEC-J2 cells treated with DON. The results showed that 2 μg/mL of DON decreased both claudin-3 and ZO-1 (Figure 1C), as evidenced by the loss of the outer-line of ZO-1 expression on DON-treated IPEC-J2 cells (Figure 1D). Therefore, treatment of DON at 2 μg/mL induced a breakdown of epithelial integrity and reduced the expression of TJ proteins, coincident with lower viability of IPEC-J2 cells.

### TLR2 ligands increased TJ barrier function and up-regulated the expression of TJ proteins on IPEC-J2 cells

**Figure 2:**
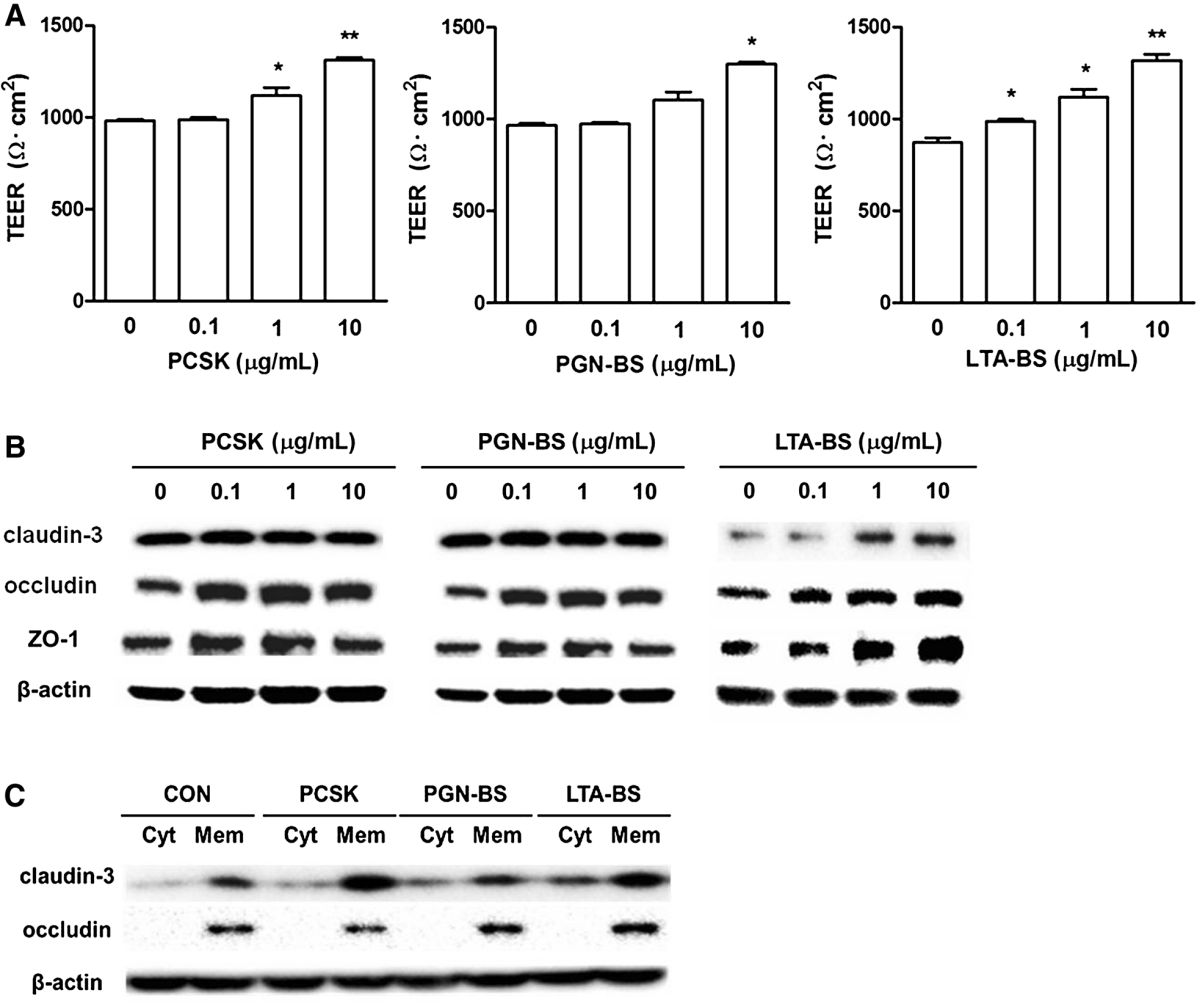
**TLR2 ligands enhanced barrier function in IPEC-J2 cells.** IPEC-J2 cells were treated with synthetic TLR2 ligands, Pam3CSK4 (PCSK), *B. subtilis*-derived PGN (PGN-BS) or *B. subtilis*-derived LTA (LTA-BS) at 0, 0.1, 1, or 10 μg/mL. **A** TEER was examined at 24 h using epithelial voltohm meter. Data are presented as mean ± SD (*n* = 4). *P < 0.05; **P < 0.01, determined by one-way ANOVA with Tukey’s posttest. **B** The monolayer of IPEC-J2 cells was lysed and protein extracts were analyzed for claudin-3, occludin, and ZO-1 by using Western blot assay. **C** Lysates were produced from membrane and cytosolic portion of the cells and the expression of claudin-3 and occludin at 24 h was examined by Western blot assay. β-actin was used as an internal control (*n* = 3). The representative figure from three similar results is shown. Cyt; cytosol, Mem; membrane.

TLR2, expressed on epithelial cells [21], is known to enhance intestinal barrier function [22]. In our previous study, we demonstrated that *B. subtilis*-derived LTA (LTA-BS)and *B. subtilis* increased barrier function in IPEC-J2 cells [15]. To investigate the TLR2-mediated effect on barrier function of porcine epithelial cells, IPEC-J2 cells were stimulated with LTA-BS, PGN-BS, *S. aureus*-derived LTA (LTA-SA), and synthetic TLR2 ligand Pam3CSK4 (PCSK) and *Escherichia coli*-derived lipopolysaccharide (LPS) as a TLR4 ligand [23]. All TLR2 ligands used in the present study significantly increased the TEER of IPEC-J2 monolayers in a dose- and time-dependent manner after 24 and 72 h treatment (Figure 2A). In contrast, LPS had minimal or no effect on barrier integrity in the current study (data not shown).

To further investigate whether the barrier-enhancing effect of TLR2 ligands is associated with TJ proteins, we examined the expression of key TJ proteins in the IPEC-J2 cells. We found that the expression of claudin-3, occludin, and ZO-1 was increased especially after LTA-BS treatment (Figure 2B). TJ proteins can be classified into membrane and cytosolic components [24]. Barrier integrity is determined by forming complex at the transmembrane regions as well as total TJ proteins [25, 26]. To validate the localization of TJ proteins, the cells were divided into cytosolic (hydrophilic region) and membrane (hydrophobic region) fractions. The results showed that the expression of TJ proteins was increased in the membrane fraction, indicating that these proteins were localized mostly in the membrane after treatment with the TLR2 ligand (Figure 2C). Collectively, these results demonstrate that epithelial cells treated with TLR2 ligand may enhance intestinal barrier function and its integrity.

### Pretreatment with TLR2 ligands led to barrier protection against DON exposure in IPEC-J2 cells

**Figure 3:**
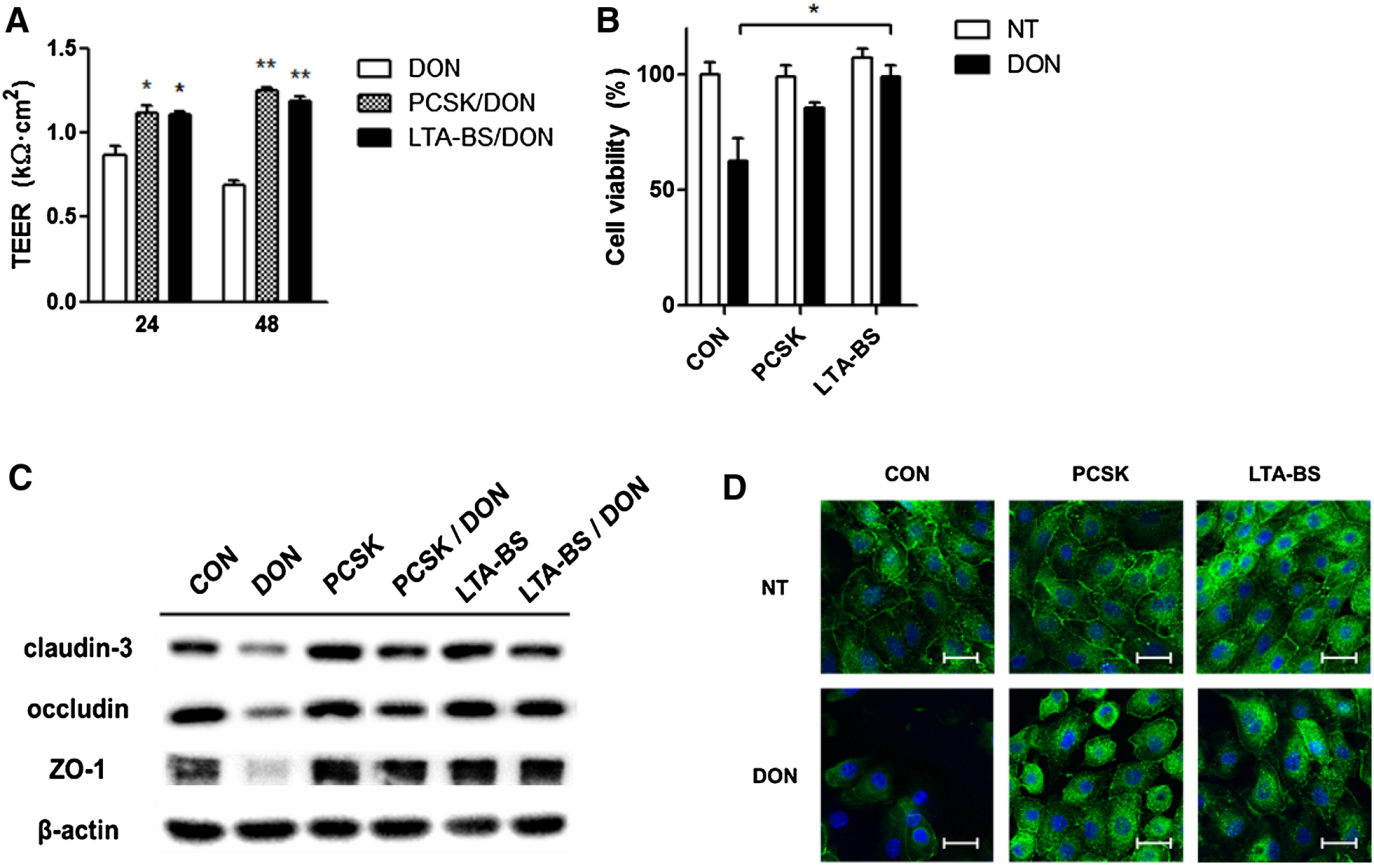
**IPEC-J2 cells treated with TLR2 ligand showed a protective effect against DON-induced damage.** IPEC-J2 cells were stimulated with 10 μg/mL of synthetic TLR2 ligand, PCSK or LTA-BS and then treated with or without DON (2 μg/mL) for 24 or 48 h. **A** TEER values were examined using epithelial voltohm meter. Data represent mean ± SD of TEER (*n* = 3). *P < 0.05; **P < 0.01, determined by one-way ANOVA with Tukey’s posttest at each time point. NT denotes no treatment. **B** Viability of the cells was examined by MTT assay at 48 h after DON treatment (*n* = 4). *P < 0.05, determined by two-way ANOVA with Bonferroni’s post. **C** Protein levels of claudin-3 and ZO-1 at 48 h after DON exposure were examined from whole-cell lysates by Western blot assay. β-actin was used as an internal control. **D** ZO-1 expression was visualized using confocal microscopy after staining with anti-ZO-1 antibody conjugated with Alexa fluor 488-FITC (green) and nuclei (DAPI; blue). The representative figure from four similar results is shown. Scale bar = 50 μm.

To investigate the prophylactic effect of TLR2 ligands against DON-induced barrier disruption, two types of TLR2 ligands (10 μg/mL of PCSK and LTA-BS) were pretreated in IPEC-J2 cells for 24 h, followed by DON treatment (2 μg/mL) for 48 h. Interestingly, the integrity of the barrier treated with TLR2 ligand was preserved, showing nearly normal values, which were similar to the TEER of the control (Figure 3A). Additionally, when the cells were treated with LTA-BS prior to DON treatment, cell viability was significantly higher than that observed with DON treatment alone (Figure 3B). Additionally, expression of GM-CSF, which is required for the survival and proliferation of epithelial cells [27], was not decreased in IPEC-J2 cells pretreated with TLR2 ligand (Additional file 2A). TJ protein expression was also higher than that observed with DON treatment (Figure 3C). Confocal microscopic analysis further revealed that TLR2 ligands induced the barrier formation of IPEC-J2 cells and protected them from DON-mediated damage (Figure 3D). MCP-1 is one of the key chemokines that regulate migration and infiltration of monocytes/macrophages [28], and contributes to increased endothelial permeability by regulating the redistribution of TJ proteins [29]. We found that DON-induced upregulation of MCP-1 expression in epithelial cells was decreased following pretreatment with TLR2 ligand (Additional file 2A). Thus, TLR2 treatment showed a barrier-protective effect by preventing DON-induced damage and sustaining TJ formation.

### Association of PI3K-Akt signaling with barrier regulation was increased by TLR2 treatment in IPEC-J2 cells

**Figure 4:**
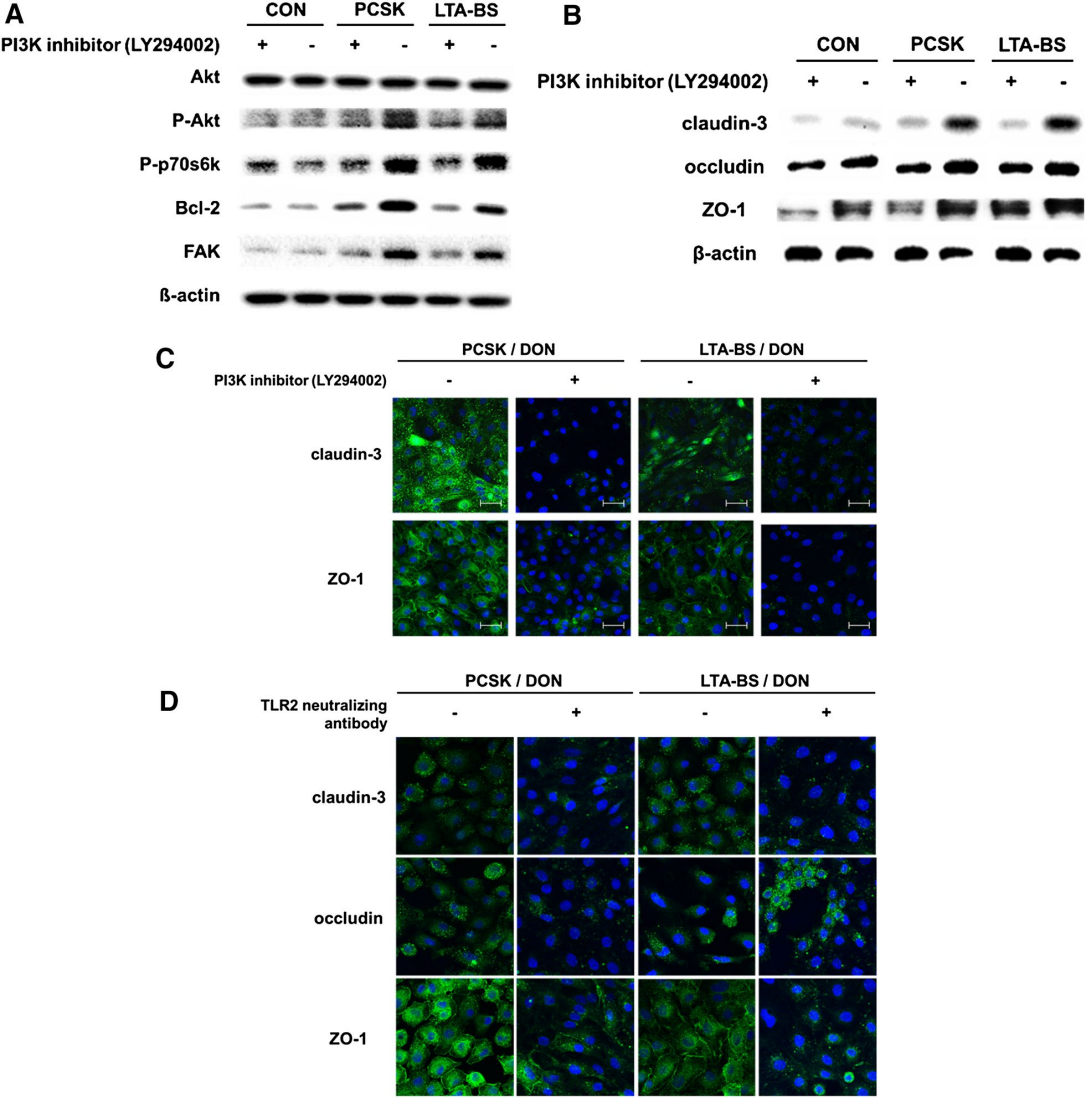
**TLR2 ligands induced PI3K-Akt-dependent regulation of intestinal barrier function in IPEC-J2 cells.** IPEC-J2 cells were stimulated with PI3K inhibitor, LY294002 (10 μg/mL) for 1 h prior to the treatment with PCSK and LTA-BS for 24 h. **A** Total Akt (serine 473), p-Akt, p70S6K, Bcl-2, FAK, and β-actin were examined using Western blot assay. **B** TJ proteins, claudin-3 and ZO-1 compared to β-actin control were examined for their expression from whole-cell lysates by Western blot assay. The representative figure from four similar results is shown. **C** The expression of claudin-3 and ZO-1 in the cells was visualized using confocal microscopy after staining with anti-claudin-3 or -ZO-1 antibody conjugated with Alexa fluor 488-FITC (green) and nuclei (DAPI; blue). The representative figure from four similar results is shown. **D** The cells were pre-treated with TLR2 neutralizing antibody (20 μg/mL) for 1 h before TLR2 stimulation in order to neutralize TLR2. DON (2 μg/mL) was treated for 48 h, and the expression of claudin-3, occludin, and ZO-1 was visualized using confocal microscopy. The representative figure from four similar results is shown. Scale bar = 50 μm.

It has been demonstrated that TLR2 enhances ZO-1-associated intestinal epithelial barrier integrity via the PI3K/Akt pathway [4]. To examine the involvement of PI3K signaling in the regulation of TJ protein-associated barrier function, we blocked the PI3K signal, and changes in PI3K-related molecules and barrier integrity were evaluated 24 h after TLR2 ligand treatment. Notably, the results showed that IPEC-J2 cells treated with TLR2 ligands, either synthetic PCSK or LTA-BS, increased the phosphorylation of Akt and p70S6K. However, phosphorylation was decreased in the presence of the PI3K inhibitor (Figure 4A), indicating that TLR2 stimulation is associated with PI3K signaling.

B cell lymphoma 2 (Bcl-2), an anti-apoptotic protein, and focal adhesion kinase (FAK), a non-receptor tyrosine kinase, play important roles in cell adhesion, proliferation, survival, and barrier function [21, 30]. We found that activation of these molecules was largely inhibited by the PI3K inhibitor (Figure 4A), suggesting that TLR2-ligand treatment could down-regulate cell death via a PI3K-associated mechanism.

Next, to investigate whether the protective effect of TLR2 ligands on barrier function against DON is associated with PI3K signaling, we blocked this signal and examined TJ protein expression in IPEC-J2 cells pretreated with TLR2 ligand and then exposed to DON. The results showed that TLR2 ligand (both PCSK and LTA-BS)-mediated expression of claudin-3 and ZO-1 was resistant to DON exposure, which was significantly suppressed by PI3K inhibition (Figures 4B and C). Thus, TLR2 signaling induced a protective function of the barrier that was related to downstream PI3K-Akt signaling.

In addition, because PCSK and LTA-BS are sensed by TLR2, we investigated whether TJ formation was also enhanced by PCSK and LTA-BS against DON in the absence of TLR2 signaling. The result showed that pretreatment with TLR2 neutralization antibody completely inhibited the PCSK- and LTA-BS-mediated barrier protective effect (Figure 4D). Furthermore, pretreatment with TLR2 ligand enhanced TLR2 expression of the epithelial cells that caused resistance against DON-induced damage (Additional file 2B), indicating that TLR2 stimulation plays an important role in TLR2-mediated barrier regulation. These results indicate that TLR2-mediated barrier function influences the expression of intestinal TJ proteins and has a critical role in the protection against DON-induced barrier damage.

### TLR2 stimulation led to cell survival and proliferation of monocytes and lymphocytes

IEC maintains close communication with immune cells in the lamina propria [20]. First, we set the co-culture system by incubating IPEC-J2 cells together with PBMCs using a trans-well plate to mimic the intestinal environment. We found that co-culture of PBMCs with IPEC-J2 cells reduced apoptosis, as shown by low Annexin V expression in PBMCs compared to that in PBMCs without co-culture (Additional file 3A), suggesting that IPEC-J2 cells had a positive impact on immune cell survival. Coincidently, co-cultured CD3^+^ lymphocytes retained better CD4 and CD8 expression, and CD172a^+^ monocytes showed increased CD163 expression at 72 h after co-culture compared to cells that were not co-cultured (Additional file 3B).

**Figure 5:**
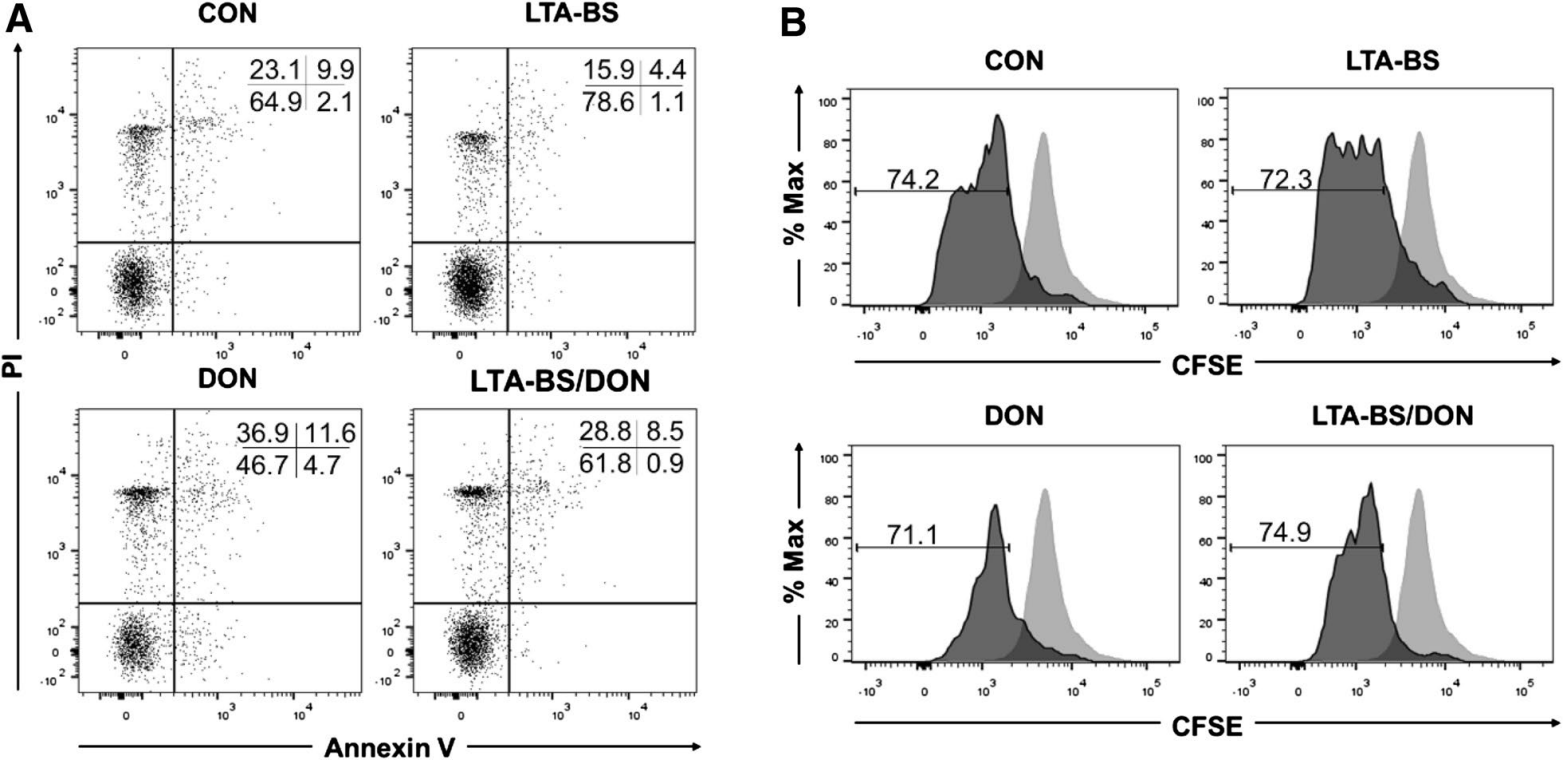
**TLR2 ligands affected the fate of innate immune cells in IPEC-J2/PBMC co-culture system.** IPEC-J2 cells and porcine PBMCs were co-cultured using trans-well plate. The cells were treated with *B. subtilis*-derived LTA (LTA-BS, 10 μg/mL) for 24 h, followed by DON treatment (2 μg/mL) for 48 h. **A** Attached cells in the bottom well were trypsinized and combined with the rest of the cells. Then, the cells were stained with anti-Annexin V and PI, and analyzed for the cell death using flow cytometry. The data represent means of the percentage of cells out of total cells ± SD (*n* = 4). **B** To investigate proliferation, porcine PBMCs were stained with CFDA-SE before co-culture. Then, the cells were treated with DON for 48 h with or without pretreatment with LTA-BS, and the cell proliferation was measured by flow cytometry. The degree of proliferation was shown as percentage (mean ± SD) of CFSE on CD172a^+^ monocytes. The representative result from four similar results is shown.

We further hypothesized that damage to intestinal epithelial cells treated with DON affected the immune cells. Therefore, we investigated the survival of PBMCs co-cultured with IPEC-J2 cells damaged by DON treatment. The results showed that CD163 expression of CD172a^+^ monocytes was diminished by DON treatment coincident with elevated tumor necrosis factor-α production (Additional file 4). Thus, DON treatment on the apical side may be involved in the inflammatory response. We also found that DON treatment also led to significantly higher expression of Annexin V (apoptotic cells) and PI (necrotic cells) of PBMCs on the basolateral side when compared to that in the control (apoptotic cells, 4.74% versus 2.12%, and necrotic cells, 36.9 versus 23.1%, respectively), coincident with the proportional decrease in live cells (46.7 vs. 64.9%) (Figure 5A). Notably, LTA-BS treatment of epithelial cells showed a reduction in apoptotic and necrotic cells in PBMCs that were co-cultured. Furthermore, TLR2 ligand pre-treatment resulted in decreased apoptosis of PBMCs against DON exposure, coincident with increased live cells compared to that in non-treated cells (apoptotic cells, 4.74 vs. 0.88%). In addition, proliferation of myeloid cells (CD172a^+^) appeared to be suppressed by DON under the same condition, while proliferation was increased after pretreatment with TLR2 ligand (Figure 5B). Thus, TLR2 treatment had a positive effect on the survival and proliferation of immune cells.

## Discussion

In the present study, we examined the mechanism by which TLR2 signaling regulates the barrier function of porcine intestinal epithelial cells exposed to DON. In IEC, TJs are multiple protein complexes that link the apical side of the epithelium, playing an important role in barrier integrity [31]. TLR2 stimulation has previously been shown to preserve TJ-associated barrier function, which is controlled by the PI3K/Akt pathway and PKC signaling [22, 32]. However, the regulation of TJs by TLR2 in pigs is not well characterized. It has been reported that TLR2 is expressed on IECs [33] and in the gut tissues [21] of pigs. Interestingly, in the present study, we found that porcine IEC showed enhanced expression of TJ proteins through PI3K/Akt signaling by TLR2 ligands, including LTA-BS, PGN-BS, and PCSK, while this was not observed with LPS, a representative TLR4 ligand.

DON is known to interfere with the expression of TJ proteins on porcine intestinal cells and binds to ribosomes to inhibit translation [34]; however, there is currently no clear strategy for protecting the porcine intestine from the toxin. Previous findings showed that *B. subtilis* can prevent IPEC-J2 cells from DON-induced barrier dysfunction [15], which led us to investigate whether prior TLR2 activation had barrier-protective properties against DON exposure. Interestingly, our results suggest that TLR2 signaling may be an effective prophylactic strategy for ameliorating damage to epithelial cells by DON in pigs. DON treatment suppressed cell viability and GM-CSF expression in IPEC-J2 cells was alleviated by (pre)exposure of TLR2 ligands demonstrated that TLR2 signaling is involved in promoting epithelial cell survival against DON. This was further supported by the observation that FAK and Bcl-2 were enhanced via the PI3K pathway. GM-CSF in the gut epithelial cell plays an important role in cell survival [27]. Moreover, FAK has been shown to regulate epithelial cell survival and proliferation during mucosal injury [30], as well as barrier function through the redistribution of TJ proteins [21]. Thus, our results suggest that the ability of IPEC-J2 cells treated with TLR2 ligands to preserve the barrier function is associated not only with modulation of the TJ assembly via the PI3K/Akt pathway, but also with promotion of epithelial cell survival via FAK and Bcl-2.

TLR2 signaling influences phynotype and/or function of immune cells directly, as demonstrated in previous studies [35, 36], as well as indirectly through IECs, as shown in the present study. Since IEC is in close contact with lamina propria cells in the intestinal tract, we hypothesized that altered barrier function, induced by apical administration of toxin, influenced immune cells on the basolateral side. Thus, we used a transwell co-culture system by incubating IPEC-J2 cells in the insert (upper part) and PBMCs in the bottom; these cells made no direct contact. We found that the presence of IPEC-J2 cells inhibited immune cell apoptosis, which was maintained better than with PBMCs alone. IECs act as modulators of the mucosal immune response by recruiting immune cells via chemokines and the induction of regulatory immune cells via various cytokines and growth factors, including interleukin-10 and transforming growth factor-β [37]. However, these factors from IPEC-J2 cells are not well defined. Using this co-culture system, we found that apical TLR2 activation alleviated apoptosis and decreased the proliferation of immune cells affected by the DON-damaged barrier. Apical TLR activation has been studied in human IECs and can drive the regulatory or inflammatory effector function of immune cells [38]. However, further studies are needed to define the effect of TLR2 ligation on mucosal effector immune responses by porcine IECs. Nonetheless, the present study suggests the utilization of a useful in vitro model for investigating the interplay between pig mucosal immune system and IECs.

This is the first study to describe the mechanism of TLR2 signaling on porcine intestinal barrier function in relation to immune regulation. We showed that (1) TLR2 activation upregulated the expression of TJ proteins on porcine epithelial cells and therefore increased barrier integrity; (2) Pretreatment with TLR2 ligands induced resistance to IPEC-J2 cells damaged by DON treatment and improved the viability of intestinal epithelial cells when cultured alone or in co-culture with immune cells; and (3) TLR2-mediated barrier function of IPEC-J2 cells was controlled via PI3K/Akt signaling. Thus, our results provide insight into TLR2 signaling in porcine epithelial cells as a potential prophylactic target for modulating gastrointestinal inflammation by promoting TJ-associated intestinal barrier function.

## Additional files

10.1186/s13567-016-0309-1 **The primer sequences for real time-PCR.** Primer sequences for real time-PCR in supplementary data (Additional files 2, 3, 4).
10.1186/s13567-016-0309-1 **IPEC-J2 cells pretreated with TLR2 ligand maintained the expression of MCP-1, GM-CSF and TLR2 against DON exposure.** IPEC-J2 cells pretreated with or without TLR2 ligand for 24 h were exposed to DON. (A) The bar graph showed the mRNA levels of porcine *mcp*-*1*, *gm*-*csf* measured using real time-PCR at 1 and 6 h after DON exposure (*n* = 3). (B) The mRNA levels of porcine *tlr2* were measured using real-time quantitative PCR analysis at 6 h. NT represents no treatment. Expression of each mRNA was presented relative to the expression of housekeeping gene, *gapdh* (*n* = 3). **P* < 0.05; ***P* < 0.01; ****P* < 0.001, determined by one-way ANOVA with Tukey’s posttest.
10.1186/s13567-016-0309-1 **Co-culture of IPEC-J2 cells with PBMCs induced positive effect on immune cells.** IPEC-J2 cells were cultured together with PBMCs on the transwell plate (0.4 μm pore). (A) Annexin V expression was measured at 48 h after co-culture (*n* = 3). * and ** indicate *P* < 0.05 and *P* < 0.01, respectively when compared to PBMCs only, determined by one-way ANOVA with Tukey’s posttest. (B) Phenotype of T lymphocyte subsets (CD3^+^ gated) and CD172a^+^ monocytes was examined by flow cytometry at 48 h after co-culture (*n* = 3).
10.1186/s13567-016-0309-1 **Apical DON treatment down-regulated CD163 expression of monocytes co-cultured with IPEC-J2 cells.** IPEC-J2 cells were cultured with PBMCs on the transwell plate (0.4 μm pore). (A) Percentages of CD163^+^ and CD163^−^ among CD172a^+^ cells were measured 72 h after 2 μg/mL of DON treatment. NT denotes no treatment. * *P* < 0.05 versus NT (*n* = 3). (B) Supernatant in basolateral side of transwell plate was collected and TNF-α was measured by ELISA at 5 days after DON treatment (0, 0.2 and 2 μg/mL) (*n* = 3). **P* < 0.05; ***P* < 0.01, determined by one-way ANOVA with Tukey’s posttest.
