# Supplementary material for: Barrier protection via Toll-like receptor 2 signaling in porcine intestinal epithelial cells damaged by deoxynivalnol
Source: Vet Res. 2016 Feb 9;47:25. doi: 10.1186/s13567-016-0309-1 (PMC4746821; doi:10.1186/s13567-016-0309-1)
Supplement: Supplementary file 1 — 10.1186/s13567-016-0309-1 The primer sequences for real time-PCR. Primer sequences for real time-PCR in supplementary data (Additional files 2, 3, 4). [file 13567_2016_309_MOESM1_ESM.docx]

**Additional file 1 The primer sequences for real time-PCR.**

| Gene | Primer sequence |
| --- | --- |
| TLR2 | Forward: 5’-TGCTGCCCAAGATTTTCGTTA-3’  Reversed:5’-TCATCACTGTGCTGGCGTTCATT-3’ |
| MCP-1 | Forward: 5’-AAGTGGGCACACCCGTTTC-3’  Reversed:5’-CGCCATTATGCGTGATTGTT-3’ |
| GM-CSF | Forward: 5’-CACTGTGGTCTGCAGCATCT-3’  Reversed: 5’-CGCTGTGTCATTACTGTTGT-3’ |
| GAPDH | Forward: 5’-TGGGCGTGAACCATGAGAA-3’  Reversed: 5’-CCTCCACGATGCCGAAGT-3’ |
