# Supplementary figures and images for: Barrier protection via Toll-like receptor 2 signaling in porcine intestinal epithelial cells damaged by deoxynivalnol
Source: Vet Res. 2016 Feb 9;47:25. doi: 10.1186/s13567-016-0309-1 (PMC4746821; doi:10.1186/s13567-016-0309-1)

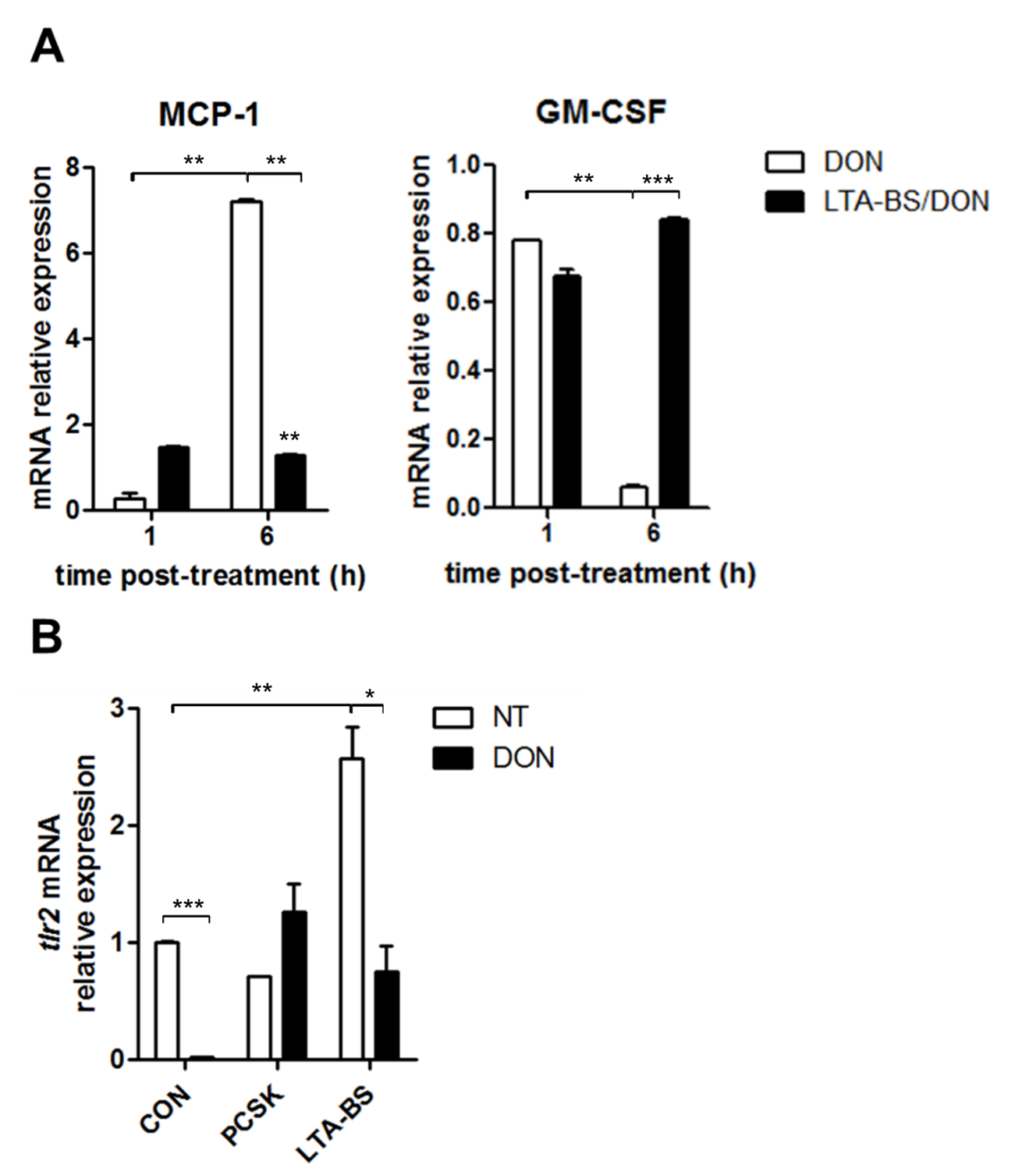

Supplement: Supplementary file 2 — 10.1186/s13567-016-0309-1 IPEC-J2 cells pretreated with TLR2 ligand maintained the expression of MCP-1, GM-CSF and TLR2 against DON exposure. IPEC-J2 cells pretreated with or without TLR2 ligand for 24 h were exposed to DON. (A) The bar graph showed the mRNA levels of porcine mcp-1, gm-csf measured using real time-PCR at 1 and 6 h after DON exposure (n = 3). (B) The mRNA levels of porcine tlr2 were measured using real-time quantitative PCR analysis at 6 h. NT represents no treatment. Expression of each mRNA was presented relative to the expression of housekeeping gene, gapdh (n = 3). *P < 0.05; **P < 0.01; ***P < 0.001, determined by one-way ANOVA with Tukey’s posttest. [file 13567_2016_309_MOESM2_ESM.docx]

**
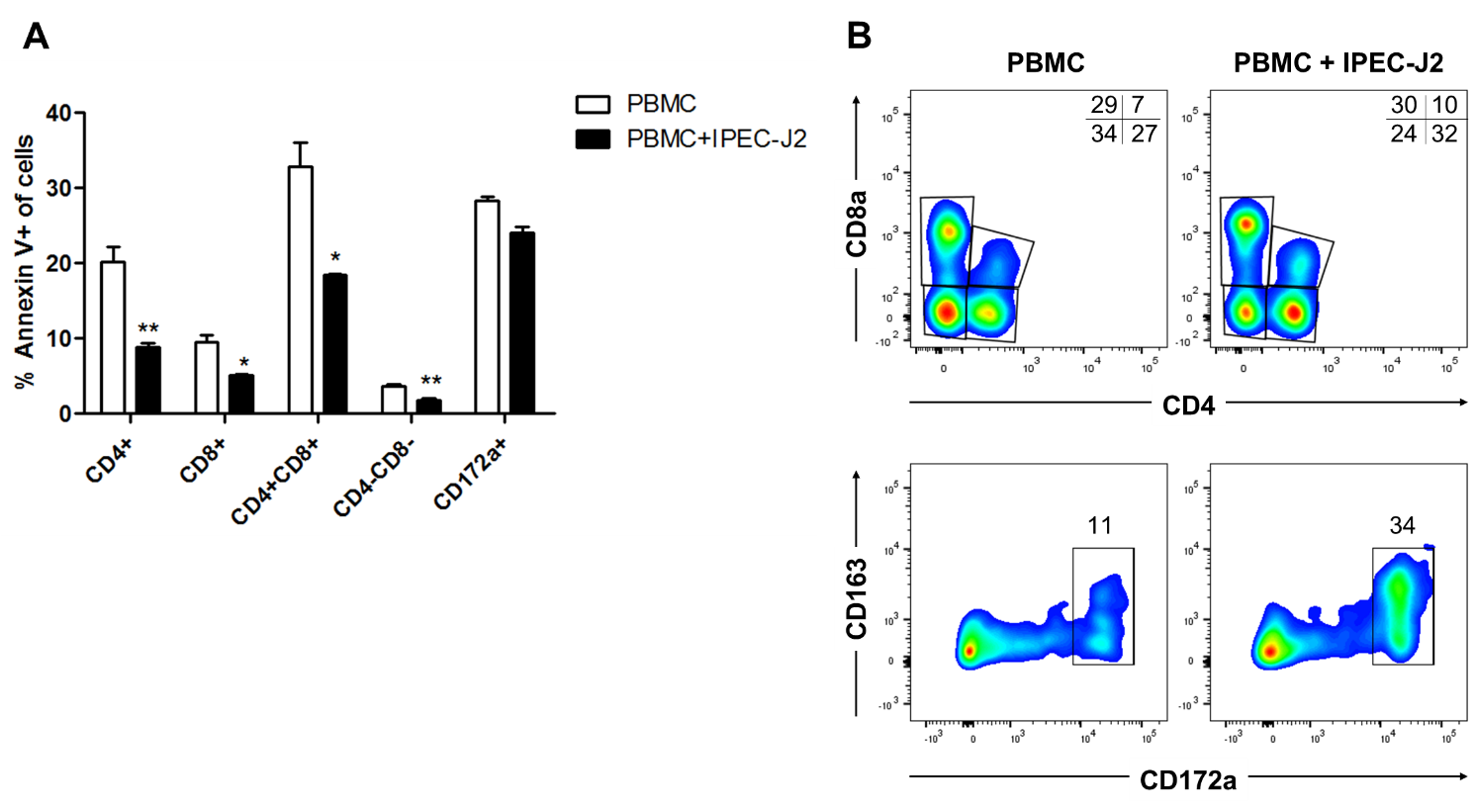
**

Supplement: Supplementary file 3 — 10.1186/s13567-016-0309-1 Co-culture of IPEC-J2 cells with PBMCs induced positive effect on immune cells. IPEC-J2 cells were cultured together with PBMCs on the transwell plate (0.4 μm pore). (A) Annexin V expression was measured at 48 h after co-culture (n = 3). * and ** indicate P < 0.05 and P < 0.01, respectively when compared to PBMCs only, determined by one-way ANOVA with Tukey’s posttest. (B) Phenotype of T lymphocyte subsets (CD3+ gated) and CD172a+ monocytes was examined by flow cytometry at 48 h after co-culture (n = 3). [file 13567_2016_309_MOESM3_ESM.docx]

**
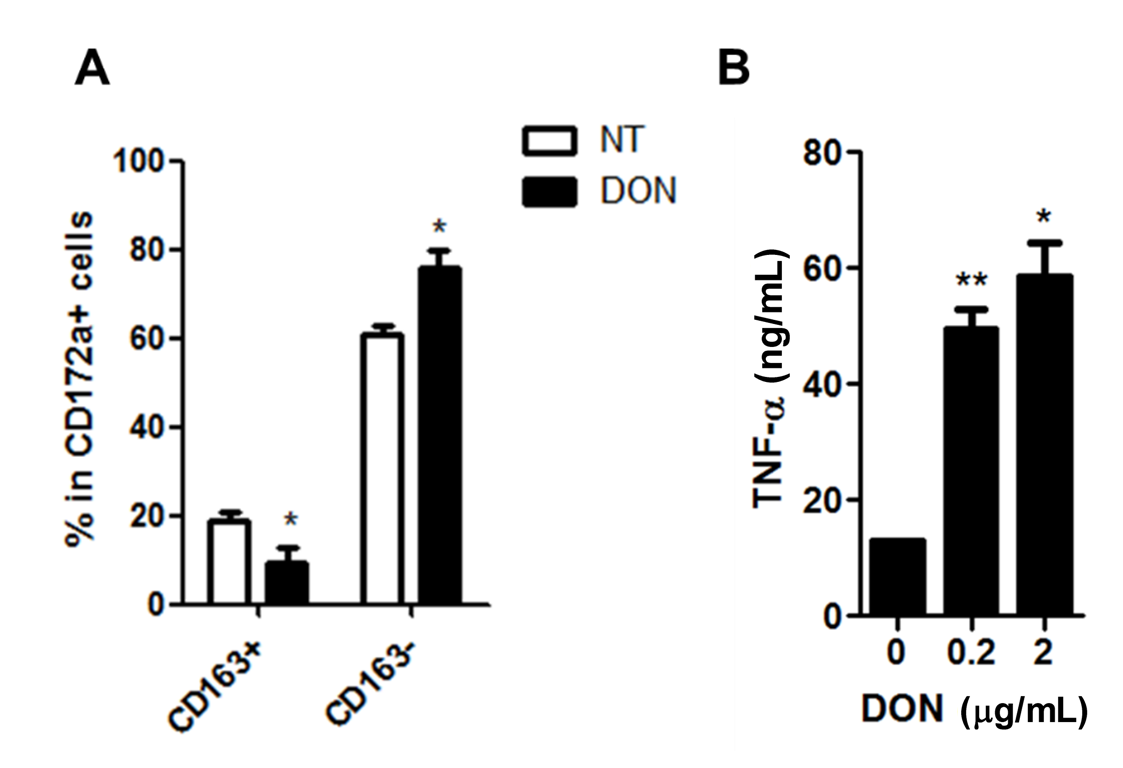
**

Supplement: Supplementary file 4 — 10.1186/s13567-016-0309-1 Apical DON treatment down-regulated CD163 expression of monocytes co-cultured with IPEC-J2 cells. IPEC-J2 cells were cultured with PBMCs on the transwell plate (0.4 μm pore). (A) Percentages of CD163+ and CD163− among CD172a+ cells were measured 72 h after 2 μg/mL of DON treatment. NT denotes no treatment. * P < 0.05 versus NT (n = 3). (B) Supernatant in basolateral side of transwell plate was collected and TNF-α was measured by ELISA at 5 days after DON treatment (0, 0.2 and 2 μg/mL) (n = 3). *P < 0.05; **P < 0.01, determined by one-way ANOVA with Tukey’s posttest. [file 13567_2016_309_MOESM4_ESM.docx]
